# The Acceptability of Using Augmented Reality as a Mechanism to Engage Children in Asthma Inhaler Technique Training: Qualitative Interview Study With Deductive Thematic Analysis

**DOI:** 10.2196/40231

**Published:** 2023-01-13

**Authors:** Antonia O'Connor, Andrew Tai, Malcolm Brinn, Amy Nguyen Thuc Hien Hoang, Daniele Cataldi, Kristin Carson-Chahhoud

**Affiliations:** 1 Respiratory and Sleep Department Women's and Children's Hospital, South Australia Adelaide Australia; 2 School of Medicine The University of Adelaide Adelaide Australia; 3 Robinson Research Institute The University of Adelaide Adelaide Australia; 4 Translational Medicine and Technology Research Group University of South Australia Adelaide Australia; 5 Australian Centre for Precision Health South Australian Health and Medical Research Institute Adelaide Australia; 6 Paediatric Medicine The Women's and Children's Hospital Adelaide Australia

**Keywords:** asthma, asthma education, pediatric asthma, augmented reality, mobile phone

## Abstract

**Background:**

Inhaled medications or inhalers provide first-line pharmacotherapeutic treatment for patients with asthma for both acute symptomatic relief and long-term management to keep symptoms under control. A good technique requires only basic instruction and training; however, a recent study identified that 92% of children do not follow all correct steps when using inhalers. There is a growing interest in technology-enhanced asthma education, with evidence demonstrating improvements in knowledge and treatment adherence. Subsequently, there are calls to explore the role of technology-based solutions in improving asthma management and disease outcomes from public health experts, health professionals, and patients with asthma. Augmented reality (AR) technology is an information delivery mechanism with proven efficacy in educational settings. AR displays digital content in a real-world environment using the camera on a smartphone or tablet device to create an immersive learning experience.

**Objective:**

The study aimed to evaluate the acceptability of AR as a mechanism for delivering asthma inhaler technique education from the perspective of children with asthma and their parents and health professionals, examined through the theoretical framework of acceptability (TFA).

**Methods:**

An asthma education resource enhanced with AR technology was created to provide inhaler technique education to children. An iterative co-design process was undertaken with target end users for a qualitative evaluation. The participants were 8 to 12 years old with asthma, their caregivers, and health professionals who had experience in managing asthma. Qualitative data were obtained through semistructured one-on-one interviews. Deductive thematic analysis using TFA was undertaken using NVivo software 2020 to assess the acceptability of AR as a delivery modality for asthma inhaler technique education.

**Results:**

Overall, 6 health care professionals, 5 asthmatic children, and 5 caregivers of children with asthma totaled a sample of 16. The use of AR in the asthma inhaler resource was found to be acceptable when responses were examined in accordance with TFA. Each of the 7 component constructs of TFA was coded throughout the 16 interviews, with *perceived effectiveness* (157 times) and *affective attitude* (63 times) coded most frequently. Positive responses included the intervention being accessible, easy to use, interesting, and fitting within the users’ value systems. Negative responses included the need to maintain an interest in children and concerns about the loss of face-to-face interaction with health professionals.

**Conclusions:**

AR appears to be an acceptable modality for delivering asthma education to children when explored using TFA constructs. Although some challenges were identified with the use of AR, the results were predominantly positive. Future designs of asthma education interventions involving AR should consider the results of this study, and further research should focus on the feasibility, usability, and barriers and facilitators of behavior change to ensure the successful implementation and uptake of AR into clinical settings.

**International Registered Report Identifier (IRRID):**

RR2-10.1177/16094069211042229

## Introduction

There are over 260 million cases of asthma worldwide, with the incidence and prevalence being higher in children than in adults [1,2]. Susceptible individuals can have symptoms of wheezing, coughing, and breathlessness [3,4]. If symptoms are poorly controlled in young people, it can lead to long-term effects, with the potential for pathological airway remodeling, impaired airway development, and possible reductions in maximal attainable lung function than those without asthma [2,5,6]. To minimize long-term airway damage, the use of inhaled medications (ie, inhalers) is the first-line treatment for both acute symptomatic relief and long-term asthma control in both children and adults [7,8]. Current guidelines state that inhaler technique education must be provided with satisfactory techniques demonstrated before the prescription of inhalers with the efficacy of education and training in improving techniques supported by a Cochrane systematic review [7,9].

Despite this, in recent studies where the asthma inhaler technique has been assessed in children, 42% of hospitalized patients have missed a critical step, and 92% of children aged 8-16 years do not properly follow all correct steps when using their inhaler [10-12]. This highlights the need to consider new approaches for educational interventions on inhaler techniques in young people. A recent nationwide survey of over 20,000 young Australians identified a 10 times greater likelihood of seeking web-based support over health professional advice to manage their stress, indicating their penchant toward technology-based solutions [13]. This preference for technology combined with the growing body of evidence suggesting that technology-delivered interventions and asthma education programs can improve knowledge, treatment adherence, and health outcomes in children with asthma highlights the need for using technology-based solutions for inhaler technique education in children to improve engagement and uptake and health outcomes [14-16]. The use of mobile technology–based solutions such as smartphones and tablet devices to deliver asthma education and self-management has also been explored in adults, with systematic reviews identifying improved quality of life and asthma control compared with routine care [17,18].

One relatively new digital solution is augmented reality (AR) defined as *technology which is able to superimpose computer-generated objects into a real-world setting so that the computer-generated objects seem to coexist in the same space in real time* and is one of the top novel technological innovations in the medical and health care industry [19,20]. They can be delivered via smartphones or tablets. It has the benefits of already having proven efficacy in other educational settings and as a behavioral change tool and would allow asthma inhaler technique education to be delivered via smartphones or tablets through videos and animations [21-26]. Given that more than 80% of children aged 5-17 years own at least one screen-based device in Australia, this suggests an appropriate and accessible delivery modality for asthma inhaler technique education [27]. Apart from 1 study showing an improved asthma inhaler technique limited by evaluation among a pediatric cohort without asthma, AR has not yet been explored in asthma education for children [28]. Research on the acceptability and awareness of this technology is paramount to informing future asthma educational interventions and their successful uptake. To address these gaps, this study aimed to evaluate the acceptability of AR as a mechanism for delivering asthma inhaler technique education.

## Methods

### Overview

An asthma inhaler technique education resource enhanced by AR technology delivered by a smartphone was co-designed for children with asthma, their caregivers, and health care professionals (HCPs) who treat asthma. Qualitative interviews based on the theoretical framework of acceptability (TFA) evaluated the acceptability of AR as a delivery mechanism. The development of TFA was described in 2017 by Sekhon et al [29] to address the lack of consistent definitions and measurements for acceptability, despite recommendations by the United Kingdom Medical Research Council that it be assessed in health care intervention development [30-33]. Acceptability has since been defined as “multi-faceted construct that reflects the extent to which people delivering or receiving a healthcare intervention consider it to be appropriate, based on anticipated or experienced cognitive and emotional responses to the intervention” [34]. Since its development, TFA has been used in multiple studies and is an accepted framework for assessing the acceptability of health care interventions [29,35-40]. A prespecified protocol for this study was published in the Australian New Zealand Clinical Trials Registry (Trial ID: ACTRN12621000306819).

### AR Intervention Development Process

To establish context for the acceptability evaluation of AR as a delivery mechanism for asthma education, a brief summary of its development is provided. The full development process will be discussed in more detail in future studies.

**Figure 1:**
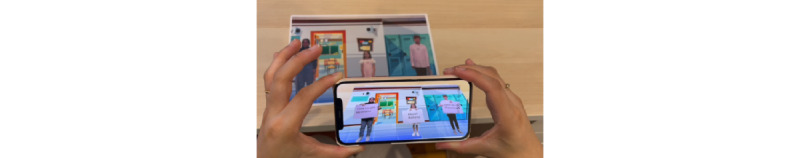
Paper-based poster triggering digital content on smartphone.

An iterative co-design process with target end users was undertaken to provide a deeper understanding of their requirements for technology use and enable improvements in the prototype asthma inhaler technique resource [41,42]. The first cohort of participants interacted with an existing cystic fibrosis–enhanced AR educational resource to provide an example of how AR functions. Their feedback was used to create an asthma-specific AR-enhanced poster to provide education on inhaler techniques. This poster was used to trigger digital educational content through the smartphone or tablet app (Figure 1). This resource was presented to the next cohort of participants who provided feedback, which was again used to enhance the intervention before being presented to the final cohort for feedback. Co-design processes optimize the uptake of digital interventions in children; therefore, this process has been used for intervention development [43-45].

### Participants and Recruitment

The participants included HCPs who managed asthma, children with asthma, and caregivers of children with asthma.

The inclusion criteria for HCPs included having worked in their profession (nursing, pediatric general medicine medical officers, general practitioners, respiratory specialists, pharmacists, and asthma educators) and having treated patients with asthma regularly for more than 12 months in the previous 5 years. The inclusion criteria in the prespecified protocol for children and adolescents with asthma were having a clinical diagnosis of asthma and being aged between 8 and 17 years. Parents and guardians of children with asthma were included if their child had a clinical diagnosis of asthma and was aged 8 to 17 years. Participants who were unable to provide consent or were non–English-speaking were excluded.

Recruitment was within a South Australian pediatric tertiary hospital, conducted by the primary investigator of the study who approached potential participants for screening. Participants were invited to participate in the study if they met the inclusion criteria and provided informed consent.

Participants were recruited from July 2021 until April 2022. Purposive sampling was intended; however, during recruitment, it became evident that this approach could not be strictly adhered to for representation across the age spectrum. This was owing to a combination of the demographics of children hospitalized for acute asthma treatment and minimization of face-to-face appointments or allowance of patients to attend hospital unless deemed medically necessary during the SARS-CoV-2 pandemic. This substantially diminished the available sample pool of older children. The inclusion criteria were changed halfway through the recruitment to children aged 8 to 12 years.

A target sample size of 15 to 20 participants was determined to achieve a large enough size to ensure sufficient breadth and depth of data but small enough to achieve meaningful analysis, which was a similar approach to other qualitative studies [46].

### Interviews and Data Collection

Qualitative data were obtained through one-on-one interviews conducted with a trained interviewer. Semistructured moderator guides were used based on TFA to aid in specifically assessing acceptability within each group of participants. The beginning of the interview explored previous experiences with asthma education, the use of smartphone and tablet apps for health, and AR. Once previous awareness and experience were assessed, the interviewer demonstrated the AR intervention to the participants and allowed them to use it themselves. Further interview questions were then asked based on the participants’ experiences of using it.

The interviews took approximately 20 to 40 minutes per participant and were audio-recorded. All the interviews were deidentified and transcribed using an automated transcription service. Interviews were check-backed and corrected by the primary investigator to ensure that they were verbatim. Transcripts were sent back to the participants to validate the content if required.

### Statistical Analysis

Using TFA as a coding framework, deductive thematic analysis was performed using the NVivo software (QSR International) [47]. Two researchers (AO and either AH or DC) jointly coded the data into NVivo to improve interrater reliability, with any disagreements resolved through discussion. The 7 component constructs of TFA consisted of an informed coding scheme. The seven constructs and their definitions are as follows: (1) affective attitude—the individuals’ feelings of the intervention; (2) burden—the amount of effort required to participate in the intervention; (3) perceived effectiveness—the extent to which the intervention is perceived as likely to achieve its purpose; (4) ethicality—the extent to which the intervention has a good fit with an individual’s value system; (5) intervention coherence—the extent to which the participant understands the intervention and how it works; (6) opportunity costs—the extent to which benefits, profits, or values must be given up to engage in the intervention; and (7) self-efficacy—the participant’s confidence that they can perform the behavior required to participate in the intervention. Affective attitude, burden, perceived effectiveness, and opportunity cost were also coded as *anticipated* or *experienced* on the basis of whether interview questions had been asked before the use of the intervention or after.

### Ethics Approval

Ethics and governance approval was obtained from the Women’s and Children’s Hospital Human Research Ethics Committee on August 21, 2020 (HREC/20/WCHN/74), and acceptance of approval was obtained from the University of Adelaide on October 20, 2021. Informed consent was obtained from all participants before the interviews were taken.

## Results

### Participant Characteristics

Of the 16 potential participants, all were recruited and analyzed. There were 6 HCPs, 5 children with asthma, and 5 caregivers (Table 1).

HCPs were split equally among medical officers and nursing staff who had treated patients with asthma for at least 12 months in the previous 5 years. Two of the participants also had a previous asthma educator role, whereas the other had a pediatric medicine educator role within the ward in which they were working. Overall, 66% (4/6) of the HCPs were female, and the remaining were male. All HCPs reported treating asthma across multiple settings, including within the community, inpatient care, outpatient care, and the emergency department.

Patients with asthma and their caregivers mostly live in metropolitan settings. All patients with asthma were diagnosed by either a respiratory specialist or a general pediatrician. Sixty percent of patients with asthma were male, and 40% were female. All caregivers were female with a range of educational levels.

**Table 1:** Participant characteristics.

Characteristics	Health care professionals (n=6)	Children with asthma (n=5)	Caregivers of children with asthma (n=5)
Age (years), mean (SD)	34.33 (4.32)	9.8 (1.10)	41.8 (2.79)
Female sex, n	4	2	5
Mean duration of asthma diagnosis (years)	N/A^a^	4.77^b^	N/A
Metropolitan vs remote, n (reference: metropolitan)	N/A	4	4
Highest level of education of caregiver (tertiary:high school)	N/A	N/A	1:1^c^
Mean duration of treating asthma for health care professionals (years)	11.68	N/A	N/A
Occupation (medical professionals:nursing:other)	1:1:0	N/A	N/A

^a^N/A: not applicable.

^b^One unknown.

^c^One participant not disclosed.

### Coding Results

#### Overview

**Figure 2:**
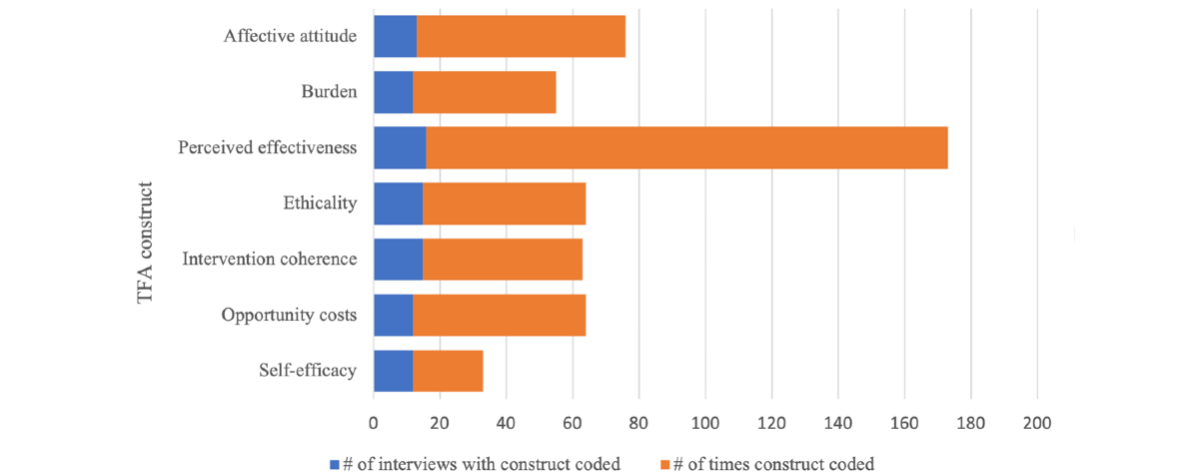
Coding frequency. TFA: Theoretical framework of acceptability.

All 7 TFA component constructs were coded throughout the 16 transcripts. The most frequently coded construct was *perceived effectiveness*, which was coded over double the number of occasions as the second most coded construct of *affective attitude*, which was coded 63 times. The remaining constructs were coded between 21 and 52 times (Figure 2).

Our findings are reported through a narrative synthesis with representative quotes for the 7 constructs of TFA. Quotes are followed by participant ID in parentheses (AC, asthmatic children; CG, caregivers of children with asthma; HP, health professionals). Table 2 expands the illustrative quotes for each construct.

**Table 2:** Theoretical framework of acceptability (TFA) construct illustrative quotes from interviews.

TFA construct	Illustrative quotes
Perceived effectiveness	Education in a different form, sort of like an interactive education or a, um, video setting, which might make it more engaging for the patient as well. [HP001] I think it’ll definitely add a component of something different and new to get them involved rather than just watching a video on a screen. [HP006] It was very informative. [PA004]
Affective attitude	Like if a kid’s looking at a piece of paper and then you put your smartphone over it and it comes to life, that’s pretty awesome. [HP002] Pretty good, actually interesting. Cause, um, it would take a lot of coding and stuff to actually work on it. [CH002] I used to think it…health apps are like boring and that stuff but virtual [augmented] reality is cool. [CH004] Um, I think it’s, it’s, uh, it’s a fantastic idea, uh, to present the information, um, in that format. Um, I think, I think the, uh, use of augmented reality is a novelty that kids would really connect with. [HP005]
Intervention coherence	Um, it was just different to how I envisioned. Um, cool that you can hover over the, the images and then it triggers where you want to learn more from. [HP003]
Ethicality	Obviously I would certainly be happy to show people how to access it and, um, you know, just show them that it is something fun and exciting to at least get them excited to then take home and, and be involved with at home. [HP006] It would be really good if it goes ahead and it’s become something that we can use…[HP002]
Self-efficacy	Pretty easy to use. [CH001] Easy to do, Just click. [PA003] Easy to use. [CH003] I think it was easy yeh. [HP006] Um, definitely good that it’s not hard to use. You kind of just go into the portal and then the videos come up, I kind of like that you have to move the phone. Um, and yeah. Seems to be pretty easy to use. [PA005]
Burden	Um, I do wonder if like, especially for littler kids, if it wouldn’t be exciting enough or interesting enough, like as an adult and probably as a teenager as well, that would be fine, but for the little ones it could potentially be boring. [HP001] Also when it does slip back to the menu, not making them sit through it again cause they’ll be like, if we want them to watch all of them. Then they’ll be like ‘oh we’ve seen this bit.' And then they’ll just lose focus. [HP004] I do wonder because um, you have, you have to hold the, uh, the, the phone or tablet up to review the video if, um, um, you know, if, if a kid’s not able to do it for that long, that might affect your, uh, ability to educate. [HP005]
Opportunity cost	Being able to actually have a hands-on with the puffer and spacer or, your airways or something like that. [HP003] You haven’t got someone there to answer your questions. So if like we, we are thinking of things and, and the feedback is there and whatever, and the parent, everyone’s individual and everyone’s got different backgrounds and different levels of understanding. And I guess the difference with education being provided here before you go home, ‘have you got any questions?’ We can answer them. [HP004] I guess that’s the only negative, is it’s not real life. Like it’s not, it is augmented reality, not reality. And so, and there are times when you get to the end of asthma education and you go, ‘you got any questions?’ And they say no, and I go, ‘so can you talk to me at what point you would come back to hospital?’ I guess you can check their knowledge rather than just assumed. [HP004] Um, it’s I guess if we, if we are using this tool, it really, it relies on, um, the family having a device that you can use. Uh, which is probably okay here in Adelaide, but I know that other places I’ve worked, um, yeah, lots of families don’t have smartphones or tablets, so yeah. I guess your uptake is limited by that. [HP005]

#### Perceived Effectiveness

Overall, perceived effectiveness from participants was positive and was coded in all interviews.

Before the intervention, all 16 participants indicated that asthma education delivered via smartphone or tablet apps or by using AR as a delivery mechanism would be a useful modality for asthma inhaler technique education (ie, anticipated effectiveness). When asked why this might be the case, patients with asthma elaborated that they *understand more with technology* [AC004], and both caregivers and HCPs described the possibility of improving accessibility to education through use of a smartphone or tablet—*It’s right there, you can just go have a look if it’s got information on it* [CG002]; *it’s something that it’s all part of our life, day-to-day* [HP003]. HCPs also described the possibility of it being a more engaging modality compared with paper-based resources:

Kids are obsessed with devices for starters, not always for a good reason, but I feel like they would, um, they would enjoy accessing this information because it is on a device, and they seem to be very device focused. Um, so it might actually capture their attention and then hopefully that would make it sink in.

HP002

After using the AR intervention, all 16 participants reported that AR delivered via a smartphone or tablet was a useful modality for asthma education (ie, experienced effectiveness). As postulated, the use of a smartphone or tablet device was described as being accessible—*something like this seems it could be much more easily incorporated into sort of home-based education* [HP001] and AR thought to add a novelty factor for children to aid in engagement—*I think it’ll definitely add a component of something different and new to get them involved rather than just watching a video on a screen* [HP006]; *It was, it was new. It was good* [CG001].

#### Affective Attitude

Affective attitude involved participants’ feelings about the use of the AR intervention. Experienced affective attitude was coded more frequently than anticipated affective attitude (55 and 8 times, respectively), which would likely reflect unknown feelings toward an intervention before experiencing it. Affective attitude was predominantly positive for both anticipated and experienced affective attitude, with participants describing AR as *cool* [AC004, HP003], *fun* [AC004] and a *great* concept [HP004]. The ability to immerse participants within the educational resource was also described:

I think it’s like, it feels more, I know that information is probably, the information that’s being delivered is the same, but you feel, feel like you’re being interacted with, rather than just...there’s the info.

HP004

One child with asthma, however, did describe having negative feelings of having too much knowledge with the use of the intervention—*...I could start worrying about my asthma. Get more worried* [AC002] and some participants had concerns over the intervention being boring—*I think they are okay. But some people might not. Just sit there and go ‘Oh this is boring, I don’t want to listen to this’* [AC002]; *I thought it was very clever. Just potentially, just boring for little ones* [HP002].

#### Intervention Coherence

Intervention coherence was defined as the extent to which participants understood the intervention and how it worked. Before the use of the intervention, many of the participants did not know what AR was; however, after experiencing the AR intervention, despite describing AR as different to what they expected—*different to how I envisioned* [HP003]; *I didn’t think people would be talking to me. I actually thought it was going to be more reading* [CG003], most understood the intended purpose of using AR and smartphone or tablet technology to improve asthma inhaler technique and increase engagement and accessibility for asthma education:

So then I could read up from the information and, you know, explain it to her and explain it to others.

HP001

I like the fact that it’s just a piece of paper, um, and they can use their own smartphone…this is so simple, like it’s just a piece of paper and your own smartphone, and I’m assuming it works with like Android or apple or whatever. So it’s really, it’s very accessible.

HP002

#### Ethicality

Ethicality described the extent to which AR and the use of smartphone or tablet technology would be a good fit within the value system of the participants. It was coded in 15 transcripts and was predominantly coded when participants were questioned about their personal views on the use of this technology in asthma education. All HCPs reported that they would use a similar intervention with AR technology if existed:

It would certainly be something that I would involve in my day to day practice if that was available to, um, show to patients, some parents.

HP006

I would, I would love to have something like this to be able to use, especially in a time pressured world. So yeah. Yeah. I’d be very happy for this to be mainstream.

HP005

All caregivers of children with asthma, and 4 out of 5 children with asthma also reported that they would use the intervention if they were available to them.

#### Self-efficacy

Self-efficacy was described predominantly when participants were discussing the ease of use of the AR intervention and access to smartphones or tablets, and it was coded in 12 transcripts. One hundred percent of children with asthma had access to a smartphone or tablet (either their own individual device or the one within the household), and most participants were confident in their own ability to use the intervention with the description of it being *easy* to use [AC001, AC003, AC004, AC005, CG001, CG002, CG003, CG005, HP003, and HP006]. All coding related to self-efficacy was positive, with no concerns raised about the difficulty or inability to use AR via a smartphone or tablet app for asthma inhaler technique education.

#### Burden

Despite the ease of use of the intervention reported by many participants in the self-efficacy construct, there was still a burden of using the AR educational intervention described above. Burden is the perceived effort required to participate in an intervention.

Burden, which was described by participants, included the inability to hold the attention of children through the educational videos alone—*It’d be good if it was more interactive* [AC002]; *I do wonder if like, especially for littler kids, if it wouldn’t be exciting enough or interesting enough* [HP002] and technical aspects of the initial iterations of the intervention—*it looked like there was a little bit of lag sometimes* [HP001]; *I guess just working out those little things, like going back to the menu or like, how do you get back to that home page?* [HP003].

The requirement of the paper-based poster required to trigger the digital educational content by holding the phone over it was also described as a burden—*It would be, it would be good if once it started playing it, you didn’t have to hold it there.* [HP004]; *if you need the paper to use the app and if patients lose the paper, then it…has some issues* [HP001].

#### Opportunity Cost

Opportunity cost, defined as the extent to which benefits, profits, or values must be given to engage in the intervention, was coded in 12 interviews. Opportunity costs were not necessarily explicitly stated, but concerns regarding parents being required to give up their values surrounding screen time if engaging in the intervention were voiced by some HCP participants—*sometimes…parents are concerned regarding screen time* and *some parents may also not like their children using a smartphone, so that might be restrictive to certain patients* [HP001]; *I think, I think there’s, um, I think there’s negatives to screens. Um, when it’s unsupervised prolonged use that becomes an addiction* [HP003]. Interestingly, this was not reported by any of the caregivers.

The concern that the use of the intervention would mean the loss of the face-to-face interaction between families and their HCPs was also expressed as was the concern that for the intervention, access to a smartphone or tablet was necessary, and so people with asthma in a lower socioeconomic status may be missed:

You haven’t got someone there to answer your questions…and I guess the difference with education being provided here before you go home, ‘have you got any questions?’ We can answer them.

HP003

Some patients and their families may not have access to a smartphone, so that provides limitations in terms of a socioeconomic point of view.

HP001

Downsides I guess, is, um, you obviously have to have access to the internet and things like that. So I suppose some disadvantaged people might not have a smartphone or wifi, et cetera.

HP006

## Discussion

### Principal Findings

This qualitative study evaluated the acceptability of AR as a delivery mechanism for asthma inhaler technique education through a robust framework of acceptability, which has been used in the evaluation of other health care interventions [29,35-40].

Overall, participants positively reported the use of AR as a delivery mechanism for asthma inhaler technique and found it to be an acceptable intervention. This is in line with other studies that have examined the acceptability of the use of digital technologies for children and adolescents with asthma who have also indicated generally positive findings regarding the acceptability of interventions [48-50].

The TFA construct of perceived effectiveness was the most coded and reported by all participants. Participants found AR to be new and interesting for children, which would allow for increased engagement in inhaler technique education and the use of smartphones and tablets as an accessible modality for many communities with asthma. In recent years, challenges from the SARS-CoV-2 pandemic have highlighted the need for alternative health care education delivery mechanisms [51-53]. Therefore, the ability of this intervention to be delivered at home or in other nonclinical settings is advantageous.

The ease of use of a digital health intervention is also important with regard to acceptability. A recent pilot study by Davis et al also discussed the importance placed by participants on the ease of use of a co-designed goal-setting asthma app for young people with asthma [44]. The ease of AR use was highlighted in the TFA construct of self-efficacy, in which participants reported simplicity of the intervention and the ability to confidently use AR technology independently via a smartphone device. Other forms of modern technologies, including virtual reality, may require additional equipment such as head-mounted displays or headphones to create a fully immersive experience, highlighting the relatively uncomplicated nature of AR as a benefit in this investigation [54-56].

The challenges to acceptability included the perceived burden of maintaining the attention of the children through educational videos alone, with suggestions such as increased gamification and animation provided by participants to try to combat this. Recently, multiple publications and systematic reviews have provided evidence of the potential of gamification to improve learning outcomes and promote positive behavioral change in various health care or educational settings [57-63]. Similarly, the impact of animation on visual attention has recently been studied in a systematic review that reported the positive influence animation has on viewers’ attention and learning skills [64]. Regarding asthma specifically, smartphone apps such as *AsthmaXcel Adventures*, which use gamification and animation have been shown to improve asthma control and knowledge and reduce morbidity such as emergency department visits in pediatric patients with asthma, strengthening the case to incorporate these into developing AR interventions [65]. Technical difficulties of the intervention and the use of the paper-based resource requirement to trigger digital content also provided challenges for the use, which in further iterations will be ironed out to minimize this as a barrier for uptake. The opportunity cost of the lack of face-to-face interaction with HCPs was also identified with the use of AR via smartphone or tablet technology. It is possible this may be overcome via incorporation of a *chat* function with HCPs within the digital intervention, such as in the mobile health app designed by Kosse et al, who also showed improved adherence to asthma medication in adolescents with asthma who used this function [66].

### Strengths and Limitations of the Study

The strengths of this study include the recruitment of likely end users for the intervention of participants to ensure optimization of information-rich data and the rigorous qualitative methodology applied to this evaluation. The gold-standard methodology included a prespecified published protocol, qualitative interview training, transcription of audio files, 2 coders to reduce interpretation bias and use of a well-established theoretical framework. The use of TFA allowed interview questions to be formed with a theoretical basis and allowed for comparison with other studies that use TFA to explore similar themes.

This study has several limitations, including generalizability, limitations of AR technology, and a purely deductive analysis. Generalizability was limited because patients with asthma were excluded if English was not their first language. This precluded the evaluation of acceptability in other ethnic backgrounds, particularly in Aboriginal and Torres Strait Islander children in Australia, who have approximately 2 times the asthma prevalence than children who are non-Indigenous [67]. Participants were also unable to use the intervention if they had any visual or hearing impairment. As mentioned in the Methods section, the recruitment inclusion criteria were also changed during the study owing to the restrictions of the COVID-19 pandemic and the diminished sample pool of older children (>12 years). To ensure that we adhered to purposive sampling, we could have increased the sample size or adjusted the parameters of our age inclusion criteria. After careful consideration and consultation with both asthma clinical care experts and experts in technological innovation design, it was decided that targeted, more meaningful information would be more likely to be obtained if only the younger cohort was included. It was also felt that the older cohort would have different design and content requirements. Therefore, we did not review the acceptability of AR in the adolescent age group but will do so in a future study. Other aspects of purposive sampling such as representation of the 3 different participant groups and gender were achieved. Purposive sampling is commonly used in qualitative research; however, there is an inherent risk of selection bias, which is another limitation of this study. Recruitment was also only undertaken at a single site—a tertiary pediatric hospital—indicating that the sample pool may not have had widely differing opinions. Patients and parents recruited may have had poorer control or more severe asthma, and HCPs may have been more experienced in managing asthma and providing education to this specific subpopulation. The AR intervention itself also had limitations owing to the availability of only a small amount of funding to design and develop the software, content, and scope of information. This may have affected the feedback received from the participants, especially in terms of the burden of use. This study also used a purely deductive approach for data analysis, which meant that there may have been data that did not fall within TFA and hence possibly missed. However, as TFA was also used as the basis for interview questions within the semistructured moderator guides, the data generated predominantly fell within the TFA constructs. We did not identify any key outliers or recurring themes outside TFA.

### Implications for Future Research and Clinical Practice

AR is a relatively novel technological innovation; only 1 previous study has explored its use as a delivery mechanism in asthma inhaler techniques in children, and no qualitative research on its acceptability has been undertaken [28]. Although there have been multiple studies evaluating the acceptability of mobile apps and other digital interventions in patients with asthma, to the best of our knowledge, this is the first study to evaluate the acceptability of AR for asthma inhaler technique education [68-70].

Our findings can inform future designs that should consider incorporating features such as gamification to further increase engagement and ensure a streamlined design with minimal technical difficulties to decrease the perceived burden of use. The possibility of including interactions with health care professionals may also be beneficial to decrease the perceived opportunity cost of loss of the ability of caregivers and children to ask questions, provide feedback, and knowledge *check back*.

To ensure successful uptake and implementation in the clinical setting and for broader generalizability, future research should focus on barriers and facilitators to change the usability of such interventions, feasibility (by focusing on areas such as practicality and efficacy testing), and exploration of the use of AR in other groups who may have suboptimal engagement in asthma inhaler technique education, such as adolescents.

### Conclusions

AR appears to be an acceptable modality for the delivery of inhaler education to children with asthma, their caregivers, and HCPs who provide care to young people with asthma. This evaluation provides important findings to inform further development, expansion, and upscaling of the AR education resource to address issues around inhaler technique education and potential beyond this specific issue. It also identified an appetite for novel technology-based health interventions to deliver best-practice self-management and education within the asthma community. The findings may also be used to inform the design of future interventions using AR-enabled smartphone or tablet apps to deliver health care education.

## Abbreviations

AR: augmented reality
HCP: health care professional
TFA: theoretical framework of acceptability
